# Characteristics of hospitalized adult patients with laboratory documented Influenza A, B and Respiratory Syncytial Virus – A single center retrospective observational study

**DOI:** 10.1371/journal.pone.0214517

**Published:** 2019-03-28

**Authors:** Regev Cohen, Frida Babushkin, Keren Geller, Talya Finn

**Affiliations:** 1 Infectious Diseases Unit, Sanz Medical Center, Laniado Hospital, Neytanya, Israel; 2 Ruth and Bruce Rappaport Faculty of Medicine, Technion, Haifa, Israel; Defense Threat Reduction Agency, UNITED STATES

## Abstract

**Introduction:**

The epidemiology, clinical features and outcomes of hospitalized adult patients with Influenza A (FluA), Influenza B (FluB) and Respiratory Syncytial Virus (RSV) have not been thoroughly compared. The aim of this study was to describe the differences between these viruses during 3 winter seasons.

**Methods:**

A retrospective observational study was conducted consisting of all the polymerase chain reaction (PCR)-based diagnoses of FluA, FluB and RSV among adults during 2015–2018, in one regional hospital. Epidemiology, clinical symptoms and outcome-related data were comparatively analyzed.

**Results:**

Between November 2015 and April 2018, 759 patients were diagnosed with FluA, FluB or RSV. Study cohort included 539 adult patients (306 FluA, 148 FluB and 85 RSV). FluB was predominant during the winter of 2017–18. RSV caused 15.7% of hospitalizations with diagnosed viral infection and in comparison to influenza, had distinct epidemiological, clinical features and outcomes, including older age (74.2 vs 66.2, p = 0.001) and higher rates of co-morbidities; complications including bacterial pneumonia (31 vs 18%, p = 0.02), mechanical ventilation (20 vs 7%, p = 0.001), and viral-related death (13 vs 6.6%, p = 0.04). FluA and FluB had similar epidemiology, clinical symptoms and outcomes, but vaccinated patients were less prone to be hospitalized with FluB as compared with FluA (3 vs 14%, p = 0.001). Paroxysmal atrial fibrillation and falls were common (8.7 and 8.5% respectively).

**Conclusions:**

FluA and FluB had similar epidemiological, clinical features and contributed equally to hospitalization burden and complications. RSV had a major impact on hospitalizations, occurring among the more elderly and sick populations and causing significantly worse outcomes, when compared to influenza patients. Vaccination appeared as a protective factor against hospitalizations with FluB as compared with FluA.

## Background

Influenza-like illness (ILI) is a significant cause of hospitalizations in every winter season, and is associated with increased morbidity and mortality directly from the viral infection, bacterial superinfection or exacerbation of preexisting medical conditions. The principal ILI-related viruses commonly diagnosed are Influenza A and B (FluA, FluB) and Respiratory Syncytial Virus (RSV) [1], [2]. Influenza viruses are associated with thousands of deaths worldwide annually. Each winter season is characterized by a different mix of concurrent circulation of two FluA subtypes (A/H3N2 and A/H1N1) and 1–2 antigenically and gentenically distinct FluB lineages (B/Yamagata and B/Victoria). The impact of FluB viruses on influenza-related morbidity and mortality is increasingly recognized[3] and during the last seasons a shift from B/Victoria to B/Yamagata predominance occurred in Europe[4] as well as in Israel[5]. The Israel Center for Disease Control (ICDC) performs national laboratory and clinical surveillance of ILI based on encounters in sentinel clinics and emergency department (ED) admissions of patients with ILI and pneumonia [6], but there are scarce data in the Israeli literature regarding the epidemiology, comparative clinical symptoms (FluA vs FluB) and outcomes of hospitalized adults with seasonal (not pandemic 2009/H1N1) influenza.

RSV is an enveloped, single-stranded RNA virus of the Paramyxoviridae family, considered to be principally a pediatric pathogen. Recently, RSV has been shown to account for 7–8% of adult hospitalizations with ILI [7–9], causing a more severe disease among the elderly, nursing homes [10], immune-compromised patients and in those with underlying cardiopulmonary diseases [7, 8]. Patients hospitalized with RSV have different epidemiological characteristics than patients with influenza [8, 9], being older, with more co-morbidities, and with graver hospitalizations’ outcomes[8]. There are practically no epidemiological nor clinical data regarding RSV infection among hospitalized adults in Israel. The ICDC reports that during the last 3 winter seasons, 9–18% of the ILI cases were RSV positive, but these figures include children and do not represent the hospitalization burden of RSV in Israel.

Considering the aforementioned lack of data, we aimed to investigate the epidemiology, clinical signs and symptoms, the rate and variety of complications and clinical outcomes of hospitalized patients who were diagnosed with viral-related ILI during the years 2015–2018 in our center.

## Methods

### Setting

This was a single center, retrospective observational trial, performed in the Sanz medical center, a 400-bed community-hospital, located in central Israel. Laboratory testing for patients hospitalized with respiratory viral infection (RVI) was done using a polymerase chain reaction (PCR)-based commercial system (Cepheid, Xpert Xpress Flu/RSV) for the diagnosis of FluA, FluB and RSV. Criteria for hospitalization were patients having risk factors for RVI complications (e.g. elderly patients, patients with previous cardiopulmonary disorders, immunosuppression) or patients with severe RVI (e.g. hypoxemia, respiratory distress, confusion, suspected bacterial superinfection). The decision for performing a PCR test was controlled by three infectious diseases specialists (R.C., F.B., T.F.), while being hospitalized with an RVI sufficed to order the test. Only patients that were presented to an infectious disease specialist were approved to perform a PCR testing.

Primary outcome was death during the index hospitalization. Secondary outcomes included length of stay and RVI-related complications.

#### Patients

Positive PCR laboratory results during 3 winters of 2015–2018 were retrieved from the molecular laboratory database. One infectious diseases specialist (R.C.) analyzed all the medical records. The following variables were extracted: age, sex, residency (home or long-term care facility (LTCF)), dates of hospitalization and discharge or death, date of the first symptom and length of time from this date to ED admission, the diagnosis at discharge and oseltamivir administration. Background medical conditions were extracted including calculated Charlson score. The current-season vaccination status was recorded, when noted in the medical file, as well as the patient-reported symptoms and complications that occurred during the index hospital stay. We defined health-care associated infection (HAI) (as opposed to community acquired) as symptoms of RVI occurring more than 48 hours from hospitalization.

#### Hospitalizations due to RVI

The total number of hospitalizations per month was retrieved in an aggregative manner from the hospital computer database. The diagnosis codes were reviewed and all ED codes that were found to be related to RVI were extracted and added to be the sum of ‘hospitalizations due to RVI’. The list of ICD9 codes of diagnoses can be found in the supplemental S1 Table. The rates of each PCR-proven viral infection were corrected to the corresponding month RVI-related hospitalization rate (number of viral diagnoses/100 RVI-related hospitalizations). RVI-related death was determined if the patient’s death occurred during the index hospitalization and was related to the viral infection or to its complications.

This study was approved by the institutional ethics committee of the Laniado medical center (0027-18-LND).

#### Statistical analyses

Patient characteristics were summarized using descriptive statistics. Differences between categorical and continuous variables were calculated using Pearson’s Chi square, Student’s t-test and Mann Whitney tests, as appropriate. One-way ANOVA was used to analyze differences among 3 groups, and the Games-Howell test was used in cases in which the Levine test for homogeneity showed that the groups were non-homogeneous. Kaplan-Meier method was used for survival analysis with the day of hospitalization serving as day 0. Differences between curves were calculated with the 2-sided Log Rank test. Cox regression was used to identify risk factors for death related to viral infection. Death and discharge from hospital were treated as competing events. Univariable Cox regression was conducted, and variables that had a p value <0.08 were introduced into a multivariable analysis in which we first included the age, then type of viral infection and then the significant variables from the univariable analysis using the backward stepwise method (Wald). All statistical analyses were performed using IBM SPSS statistics version 25 for Windows. In all statistical analyses, a two-sided p-value less than 0.05 was considered statistically significant.

## Results

### Patients with laboratory-confirmed RVI

**Fig 1:**
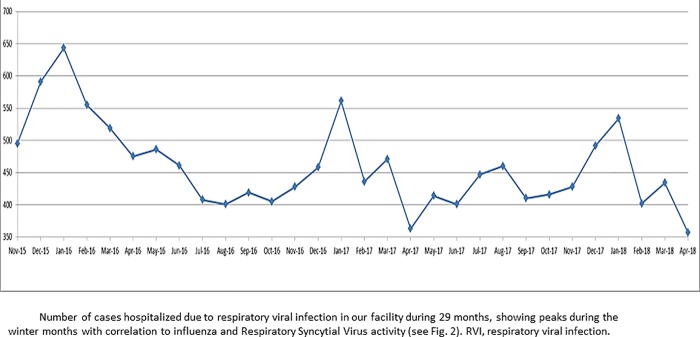
Number of hospitalizations due to RVI in 3 winter seasons.

**Fig 2:**
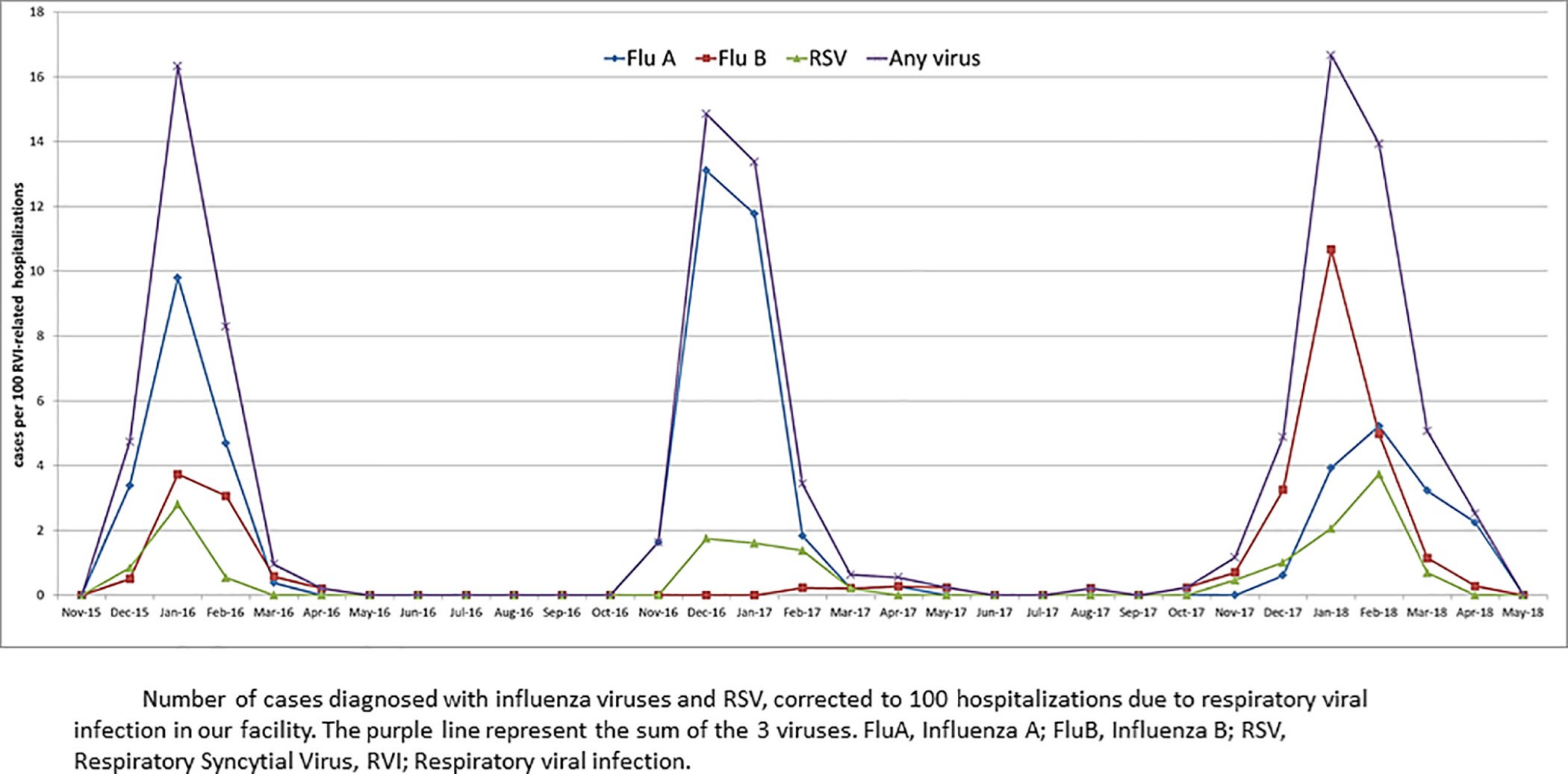
**Influenza A, B, RSV and any of the 3 viruses, corrected to 100 RVI-related hospitalizations in 3 winter seasons**.

**Fig 3:**
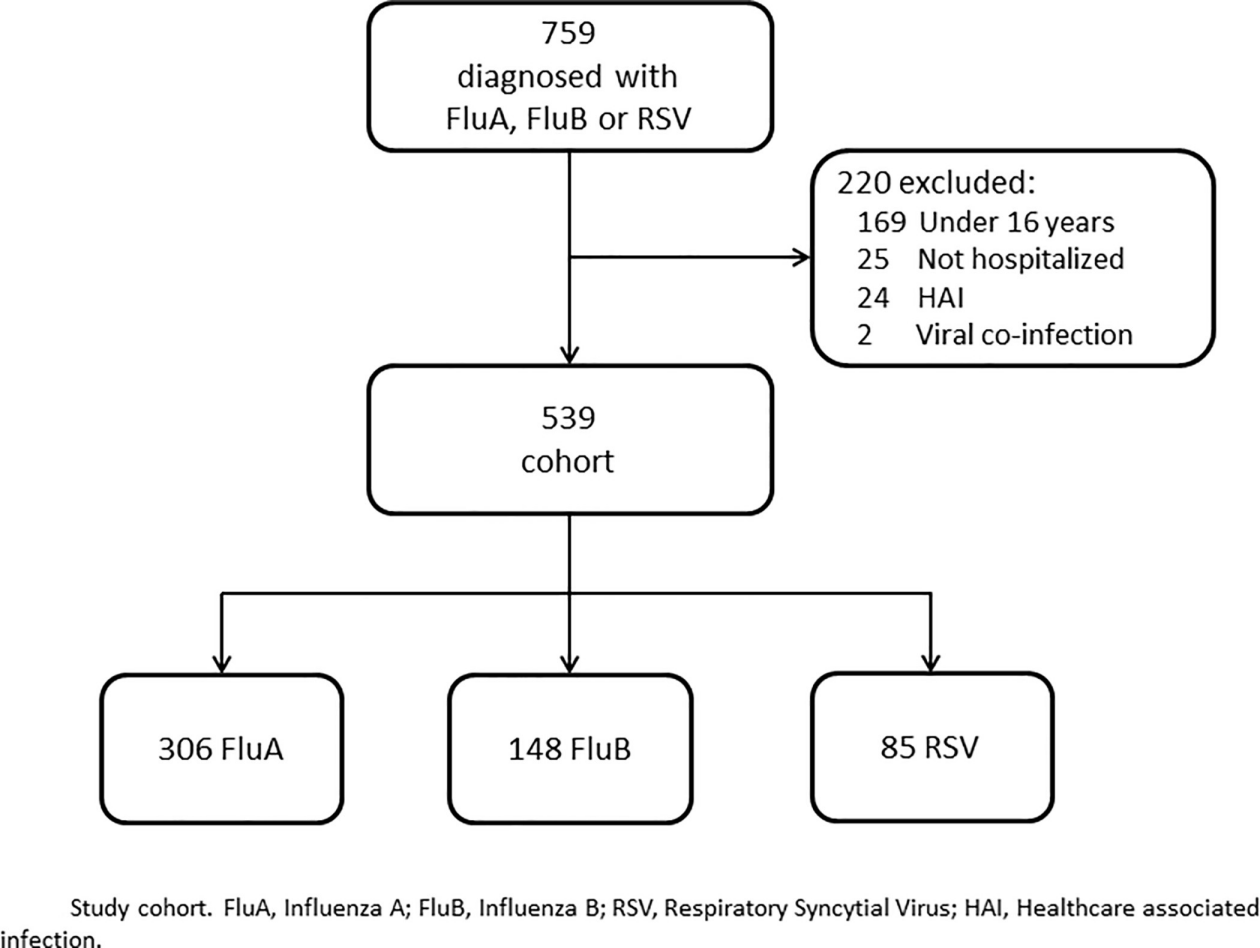
Study cohort.

Between November 2015 and April 2018, 28,722 patients were admitted to the ED with RVI, of which 13,770 were hospitalized. We identified 759 patients with laboratory-confirmed FluA, FluB or RSV. Fig 1 depicts RVI-related hospitalization and Fig 2 the number of PCR-positive cases of FluA, FluB, RSV and any of these 3 viruses, corrected to 100 RVI-related hospitalizations. During the peak winter months (December and January), positive cases with any one of the 3 viruses represented about 15% of RVI-related hospitalizations. Two hundred twenty patients were excluded: 169 were under 16 years old, 25 were not hospitalized, 24 had HAI and 2 had more than 1 virus detected. The cohort included 539 patients: 306 with FluA, 148 with FluB and 85 with RSV (Fig 3). For the analysis of signs and symptoms we included the 25 patients that were not hospitalized. In 532 out of 539 cases (98%) the PCR test was sent from the admitting ward, while 7 were diagnosed in the ED and then hospitalized.

### Seasonal viral epidemiology

In comparison with the two previous winter seasons, the 2017–2018 winter was predominated by FluB peaking earlier and higher than FluA. FluB was diagnosed in 100 patients—49% of all laboratory-confirmed viral infections in this winter (as opposed to 4 and 44 cases during the 2016–17 and 2015–16 winter seasons, respectively, Fig 2). RSV caused 15.7% of hospitalizations in patients with laboratory-confirmed RVI, being slightly more frequent in the 2017–18 season, with 36 cases (18% of RVI) identified as compared with 24 (14%) and 25 (14%) cases diagnosed in the two previous seasons (differences not reaching statistical significance).

### Patients’ characteristics

FluA and FluB had the same demographic characteristics and background morbidity, except for the incidence of ischemic heart disease (IHD) which was higher in patients with FluA as compared to those with FluB (23 vs 13%, p = 0.016), and the mean Charlson comorbidity score (higher in FluA, 4.3 vs 3.7, p = 0.03). Another exception was the vaccination history: only 3% of hospitalized patients with FluB received the inactivated influenza vaccine preceding the current winter season, as compared with patients hospitalized with FluA or RSV (14% and 12% respectively, p = 0.001), Table 1.

**Table 1:** Demographic and hospitalization data of patients with confirmed viral infection.

Variable		Flu A (n = 306)	Flu B (n = 148)	RSV (n = 85)	P value (95% CI)
Age, years, Mean (range)		66.7 (17–100)	65.1 (16–104)	74.2 (20–103)	0.004† <0.0001 (-12.7 to -3.1)††
Sex, female (%)		155 (51)	74 (50)	49 (57)	NS
Residency, LTCF (%)		34 (11)	17 (11)	13 (15)	NS
Winter season	2015–2016 (%)	102 (60)	44 (26)	25 (14)	<0.0001†
	2016–2017 (%)	137 (83)	4 (2)	24 (14)	
	2017–2018 (%)	67 (33)	100 (49)	36 (18)	
Symptoms to ED, days, Mean (range)		2.7 (0–18)	3.3 (0–29)	3.4 (0–21)	NS
LOS, days, Mean (range)		5.5 (0–63)	5 (0–49)	7.3 (0–56)	0.07^†, 0.06^†† (-4 to 0.1)
**Background morbidity**					
Diabetes mellitus (%)		84 (27)	45 (30)	37 (43)	0.018†, 0.006††
CNS disease (%)		66 (21)	26 (17)	16 (19)	NS
IHD (%)		71 (23)	20 (13)	22 (26)	0.028†, 0.016*
CHF (%)		50 (16)	17 (11)	24 (28)	0.004†, 0.002††
RHD/Valvular disease (%)		7 (2)	5 (3)	8 (9)	0.009†, 0.002††
Asthma (%)		17 (5)	8 (5)	6 (7)	NS
COPD (%)		85 (28)	35 (23)	26 (30)	NS
CRF (%)		46 (15)	13 (9)	23 (27)	0.001†, 0.001††
Hematologic disease (%)		17 (5)	9 (6)	7 (8)	NS
Immunosuppression (%)		28 (9)	13 (9)	10 (12)	NS
Chronic anemia (%)		62 (20)	29 (19)	21 (25)	NS
Previous hospitalization d/t RVI (%)		22 (7)	16 (11)	17 (20)	0.002†, 0.001††
Dementia (%)		47 (15)	24 (16)	26 (30)	0.004†, 0.001††
Active malignancy (%)		15 (5)	4 (3)	4 (5)	NS
Overweight (%)		62 (20)	27 (18)	19 (22)	NS
Pregnancy (%)		26 (8)	9 (6)	3 (3)	NS
Post labor (%)		10 (3)	4 (3)	2 (2)	NS
Charlson comorbidity score, Mean (range)		4.3 (0–16)	3.7 (0–10)	5.8 (0–11)	<0.0001† <0.0001†† (-2.3 to -0.9) 0.03* (0.03–1.14)
Current season influenza vaccine administration (%)		42 (14)	5 (3)	10 (12)	0.001†, 0.002*

†when comparing all 3 groups

††when comparing influenza A and B to RSV

*when comparing influenza A to influenza B

^not reaching statistical significance

LTCF–Long-term care facility, LOS–Length of stay, ED–Emergency Department, CNS–Central Nervous System, IHD- Ischemic Heart Disease, CHF–Congestive Heart Failure, RHD–Rheumatic Heart Disease, COPD–Chronic Obstructive Pulmonary Disease, CRF–Chronic Renal Failure, ILI–Influenza-like Illness, d/t–due to, CI–confidence interval, NS–not significant.

On the other hand, patients with confirmed RSV infection were older compared with patients with FluA and FluB (mean age 74.2 vs 66.7 and 65.1 respectively, p<0.0001). They also had more co-morbidities, with statistically significant higher rates of diabetes mellitus, IHD, congestive heart failure (CHF), rheumatic or other valvular heart disorder, chronic renal failure (CRF), dementia, and record of previous hospitalizations due to RVI. The Charlson comorbidity score was also significantly different between all the groups (RSV higher than FluA, which in its turn was higher than FluB).

### RVI characteristics

#### Influenza symptoms

The leading clinical symptoms of influenza (both FluA and FluB) were fever (82%), cough (75%), dyspnea (41%), weakness (31%), rhinorrhea (30%), vomiting (14%), chest pain (11%), chills (11%), myalgia (10%) and headache (9.6%). Other less commonly reported symptoms (<10%) were confusion, fall, syncope or pre-syncope, dizziness and diarrhea. Paroxysmal atrial fibrillation (PAF) was reported upon admission in 8% of patients with influenza.

Patients with FluB had significantly higher rates of rhinorrhea (37 vs 26%, p = 0.016) and diarrhea (9 vs 4%, p = 0.028) than patients with FluA, while patients with FluA had higher rates of dyspnea (45 vs 35%, p = 0.035) and stupor (6 vs 2%, p = 0.04), Table 2.

**Table 2:** Presenting symptoms on admission of influenza A, B and RSV.

Variable	Flu A (n = 322)	Flu B (n = 156)	RSV (n = 86)	P value (95% CI)
Fever (%)	269 (83)	128 (82)	47 (55)	<0.0001†, <0.0001††
Maximal temperature ^o^C, Mean(range)	38.6 (33.9–41)	38.6 (37.5–40)	38.5 (37.9–40)	NS
Cough (%)	249 (77)	113 (72)	62 (72)	NS
Dyspnea (%)	144 (45)	54 (35)	57 (66)	<0.0001†, <0.0001††,0.035*
Weakness (%)	94 (29)	55 (35)	17 (20)	0.04†, 0.033††
Rhinorrhea (%)	85 (26)	58 (37)	21 (24)	0.03†, 0.016*
Vomiting (%)	45 (14)	22 (14)	8 (9)	NS
Chills (%)	36 (11)	20 (13)	8 (9)	NS
Sore throat (%)	37 (11)	22 (14)	6 (7)	NS
Chest pain (%)	33 (10)	19 (12)	10 (12)	NS
Myalgia (%)	31 (10)	20 (13)	5 (6)	NS
Headache (%)	29 (9)	17 (11)	1 (1)	0.026†, 0.009††
Paroxysmal atrial fibrillation (%)	29 (9)	9 (6)	9 (10)	NS
Fall (%)	28 (9)	16 (10)	2 (2)	0.032††
Syncope/pre-syncope (%)	24 (7)	15 (10)	3 (3)	NS
Abdominal pain (%)	21 (6)	12 (8)	7 (8)	NS
Dizziness (%)	20 (6)	12 (8)	1 (1)	0.044††
Stupor (%)	20 (6)	3 (2)	10 (12)	0.008†, 0.013††,0.04*
Confusion (%)	19 (6)	15 (10)	2 (2)	NS
Diarrhea (%)	13 (4)	14 (9)	5 (6)	0.028*
Dehydration (%)	11 (3)	6 (4)	4 (5)	NS
Hoarseness (%)	5 (2)	1 (1)	0	NS
Hemoptysis (%)	4 (1)	4 (3)	2 (2)	NS
Seizures (%)	3 (1)	2 (1)	1 (1)	NS

†when comparing all 3 groups

††when comparing influenza A and B to RSV

*when comparing influenza A to influenza B

NS–not significant

#### RSV symptoms

The leading symptoms were cough (72%), dyspnea (66%), fever (55%), weakness (20%), rhinorrhea (24%), chest pain (12%), stupor (12%) and PAF (10%). Less common symptoms (<10%) included gastrointestinal symptoms (vomiting, abdominal pain and diarrhea), chills and dehydration. Patients with RSV had lower rates of fever than patients with influenza (55 vs 82%, p<0.0001), but patients already presenting with fever had a similar mean temperature compared to those with influenza. Compared to patients with influenza, patients with RSV rarely complained of headache (1%). They also had higher rates of dyspnea and stupor and significantly lower rates of rhinorrhea, confusion, dizziness, falls and weakness.

### Hospitalization outcomes

#### Disease complications

The most common complication of FluA and FluB, occurring on admission (or within 2 days), was bacterial pneumonia (19%). Any bacterial complication occurred in 27% of patients with influenza. Other common complications were cardiac and rhythm-related (22% of patients with influenza). None of the complications were found to be significantly different between FluA and FluB. Over 70% of patients with influenza were treated with antimicrobials, Table 3.

**Table 3:** Complications on admission and during hospitalization.

Variable (%)	Flu A (n = 306)	Flu B (n = 148)	RSV (n = 85)	P value
**Complications on admission**				
**Bacterial complications**				
Pneumonia	61 (20)	28 (19)	26 (31)	0.07†^, 0.02††
Pneumonia related to aspiration	12 (4)	4 (3)	4 (5)	NS
Positive sputum culture on admission	35 (11)	22 (15)	19 (22)	0.03†, 0.01††
BSI on admission	6 (2)	6 (4)	3 (4)	NS
Meningitis	2 (0.7)	1 (0.7)	0	NS
Sinusitis	2 (0.6)	3 (2)	2 (2)	NS
Urinary tract infection	4 (1)	2 (1)	2 (2)	NS
Empyema	0	1 (0.6)	1 (1)	
**Any bacterial complication**	**77 (25)**	**46 (31)**	**40 (47)**	**0.001†, <0.0001††**
Antimicrobial therapy on admission	236 (77)	102 (69)	75 (88)	0.003†, 0.006††
**Cardiovascular and rhythm complications**				
CHF exacerbation	26 (8)	8 (5)	12 (14)	0.07†^, 0.04††
Paroxysmal atrial fibrillation	29 (9)	9 (6)	9 (10)	NS
Pleural effusion	26 (9)	14 (10)	13 (15)	0.06††^
Acute myocardial infarction	17 (6)	3 (2)	3 (3)	NS
AV block	2 (0.6)	0	0	NS
Takatzubo syndrome	1 (0.3)	0	1 (1)	NS
Acute myocarditis	3 (1)	0	0	NS
Acute pericarditis	2 (0.7)	0	0	NS
**Any cardiac or rhythm complication**	**76 (25)**	**25 (17)**	**25 (30)**	**0.06†^**
**Neuromuscular complications**				
Rhabdomyolysis	7 (2)	8 (5)	0	0.03†, 0.08††^
Encephalitis	3 (1)	0	0	NS
Guillain-Barre syndrome	1 (0.3)	0	0	NS
Fracture after fall on admission	3 (1)	3 (2)	0	NS
Brain hemorrhage	1 (0.3)	0	0	NS
Acute neuralgia	1 (0.3)	0	0	NS
**Any neuromuscular complication**	**14 (5)**	**11 (7)**	**0**	**0.03**†**, 0.02**††
**Other complications**				
Acute kidney injury	53 (17)	18 (12)	14 (16)	NS
Premature uterine contractions	2 (0.7)	1 (0.7)	1 (1)	NS
Abortion	0	0	1 (1)	0.02††
Viral pneumonia	3 (1)	0	0	NS
**Complications during hospitalization**				
Acute respiratory failure	25 (8)	12 (8)	16 (20)	0.01†, 0.002††
Mechanical ventilation	22 (7)	11 (7)	16 (20)	0.003†, 0.001††
Tracheostomy	5 (2)	2 (1)	6 (7)	0.01†, 0.002††
Ventilator associated pneumonia	9 (3)	2 (1)	4 (5)	NS
Viral related death	22 (7)	8 (5)	11 (13)	0.04††
Overall death	26 (8)	11 (7)	13 (15)	0.03††

†when comparing all 3 groups

††when comparing influenza A and B to RSV

^not reaching statistical significance

BSI–blood stream infection, AV- atrioventricular, CHF–congestive heart failure, ILI–influenza like illness, NS–not significant

Similar to patients with influenza, the main complication of RSV was bacterial pneumonia (31%). Several complications were significantly higher in RSV than in patients with influenza, such as pneumonia and any bacterial complication, CHF exacerbation, acute respiratory failure, mechanical ventilation, need for tracheostomy and death related to viral infection (13 vs 6%, p = 0.04). Rhabdomyolysis, as well as other neuromuscular complications, were not seen at all among patients with RSV, in contrast to patients with influenza (0 vs 5.5%, p = 0.02). Antimicrobial therapy was administered to 88% of RSV patients, significantly more than patients with influenza (p = 0.006).

Patients with RSV had longer hospitalization duration compared to patients with FluA and FluB (mean 7.3 vs 5.5 and 5 days, respectively, not reaching statistical significance, p = 0.07).

### Antimicrobial therapy

Antibiotic therapy was administered to 77% (413/539) of the patients. In 133 of them (32%) there was a concomitant bacterial infection (115 community acquired pneumonia, 4 non-pneumonic blood stream infection, 6 sinusitis and 8 had another bacterial infection). For the rest 280 patients—in 104 patients (37%) the antibiotics were stopped when the PCR results returned, and in the remaining 176 it was continued. Most of these patients were either considered to have a concomitant atypical bacterial infection, or were very sick or fragile.

The most common bacteria that were found in the blood stream were Streptococci, followed by Gram negative non-fermenter bacilli. The bacteria that were cultivated from the blood stream and from the sputum are listed in supplemental S2 Table.

Most patients with influenza (421/454, 93%) were treated with oseltamivir.

### Patient’s survival

**Fig 4:**
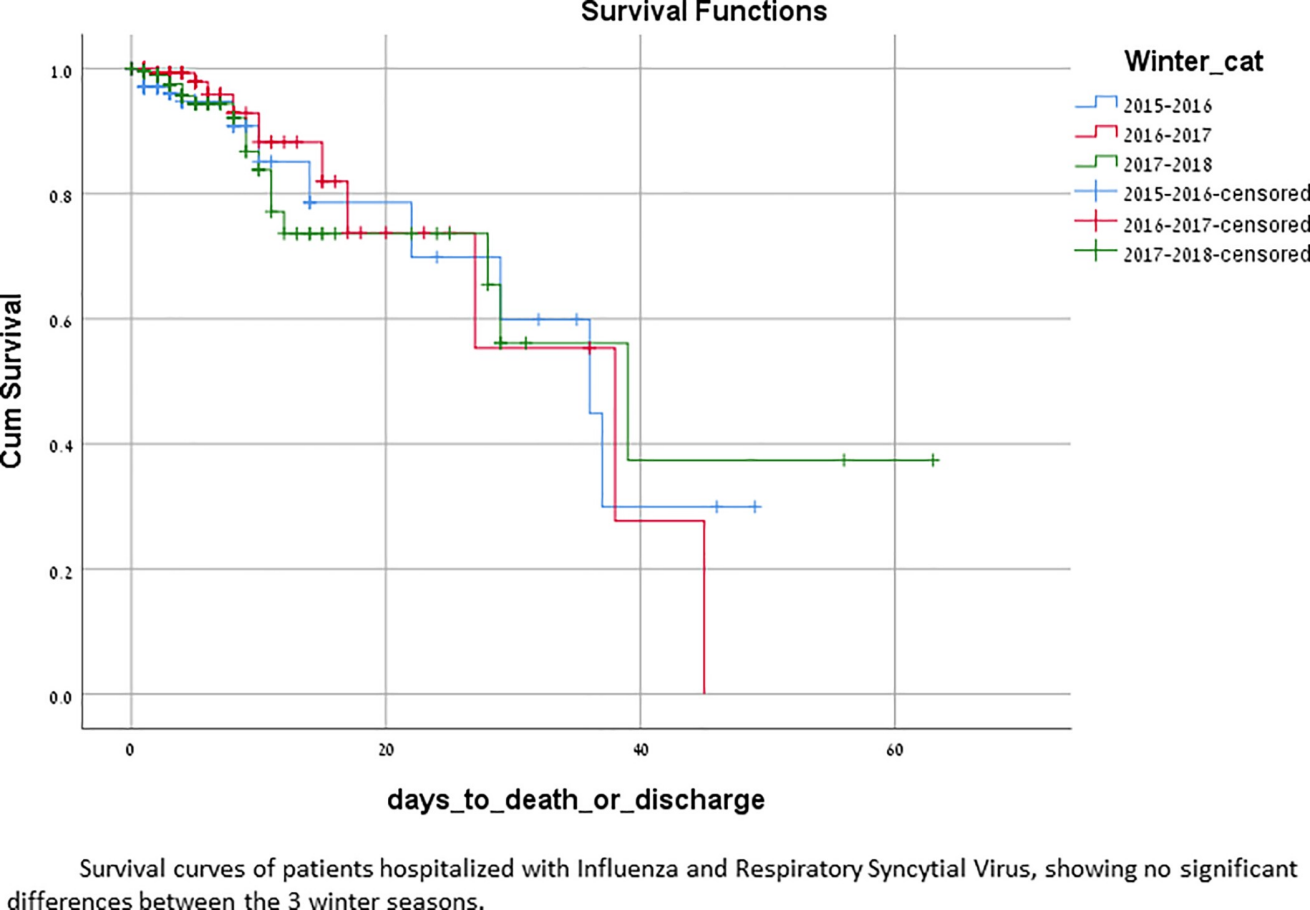
Kaplan-Meier survival analysis of patients with documented viral infection, during 3 winter seasons.

The overall RVI-related in-hospital mortality rate was 7.6%, with no significant difference among the 3 winter seasons (Fig 4).

**Fig 5:**
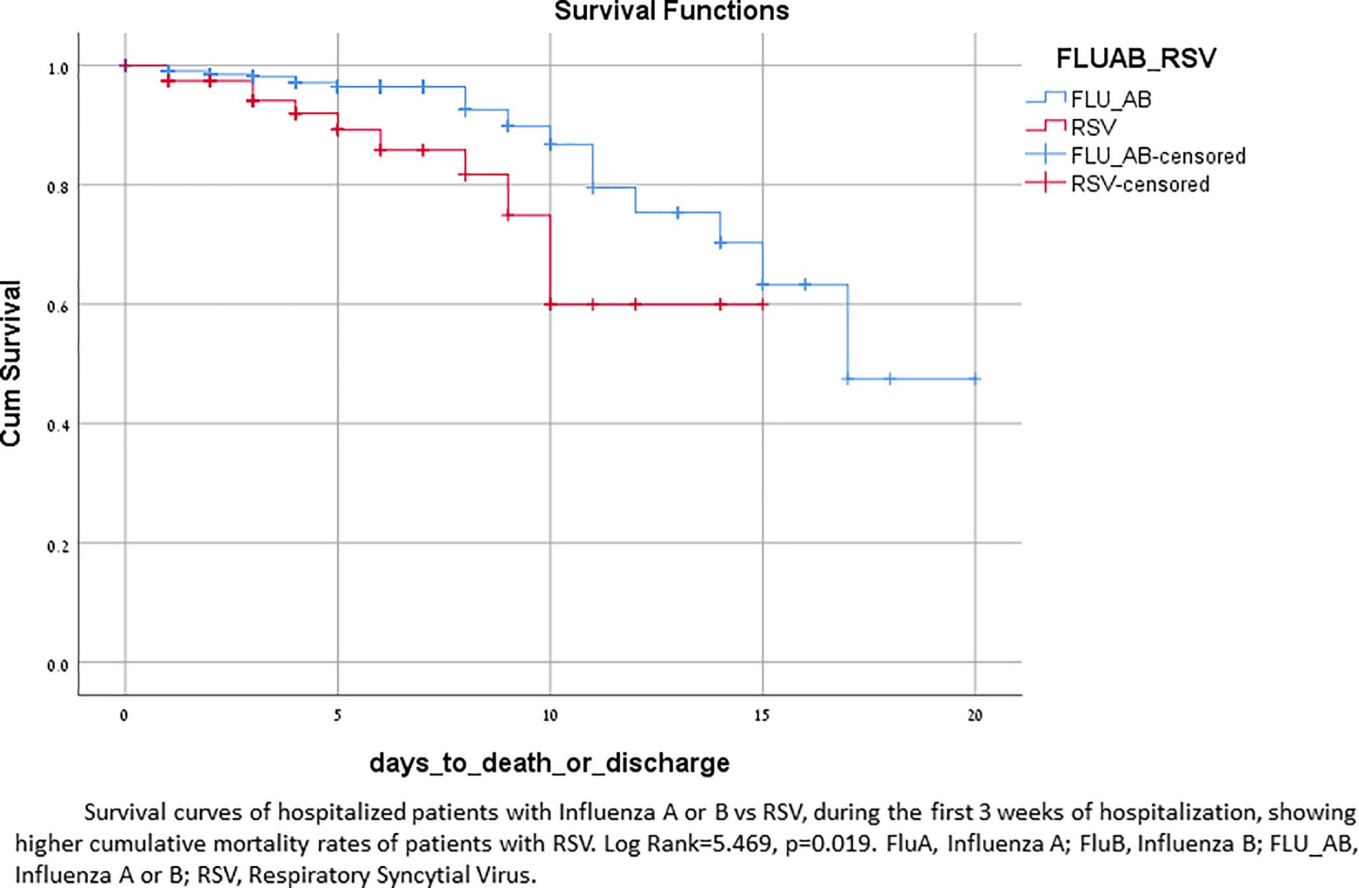
Kaplan-Meier survival analysis of patients with FluA and FluB vs RSV, during the first 21 days of hospitalization.

Death related to the viral infection or its complications was higher among patients with RSV (13%) as compared with FluA (7%) and FluB (5%). Cumulative 21-day mortality was higher in patients with RSV compared to patients with influenza, Log Rank = 5.469, p = 0.019 (Fig 5). Death rates were not significantly different between influenza patients who were treated with oseltamivir, as compared with those who were not (6.4% vs. 9.1%, p = 0.47).

The following variables on Cox regression, were found to be associated with increased risk for death: age (Hazard ratio (HR) = 1.033, p = 0.038), previous hematologic disease (HR = 4.4, p = 0.005), as well as pneumonia upon admission (HR = 3.8, p = 0.002), CHF exacerbation (HR = 4.7, p<0.0001), acute kidney injury (HR = 2.4, p = 0.016), acute respiratory failure, mechanical ventilation and aspiration pneumonia during hospitalization, Table 4.

**Table 4:** Univariable and multivariable Cox regressions of variables related to death from viral disease.

	Univariable analysis		Multivariable analysis	
Variable	Hazard ratio	p value	Hazard ratio	p value (95% CI)
Age	1.052	0.000	1.033	0.038 (1.002–1.066)
Chronic renal disease	3.3	0.000		
Previous hematologic disease	2.4	0.07	4.4	0.005 (1.5–12.6)
Active malignancy	3.5	0.02		
Charlson score	1.2	0.001		
Pneumonia on admission	4.4	0.000	3.8	0.002 (1.6–9)
Pleural effusion	2.2	0.015		
Blood stream infection	2.2	0.053		
CHF exacerbation	3.7	0.000	4.7	0.000 (2.1–10.2)
Acute kidney injury	3.7	0.000	2.4	0.016 (1.1–4.7)
Aspiration pneumonia during stay	3.2	0.001	2.4	0.045 (1.02–5.6)
Acute respiratory failure	4.7	0.000	4.1	0.02 (1.2–13.7)
Fracture on admission	4.5	0.039		
Mechanical ventilation	3.3	0.015	3.7	0.041 (1.05–13.3)

CI–confidence interval, CHF–congestive heart failure

The variables that were included in the univariable Cox analysis were: age, viral infection (FluA, FluB, RSV), sex, long-term care facility residence, background illnesses (diabetes, central nervous system disease, ischemic heart disease, congestive heart failure, rheumatic/valvular heart disease, asthma, chronic obstructive pulmonary disease, emphysema, bronchiectasis, chronic renal disease, chronic liver disease, previous hematologic disease, immunosuppression, chronic anemia, history of previous hospitalizations due to influenza or pneumonia, dementia, active malignancy, overweight, Charlson score, influenza vaccination status and complications occurring during hospitalization (stupor, fall, seizures, paroxysmal atrial fibrillation, confusion, pneumonia on admission, pleural effusion, empyema, blood stream infection, encephalitis, meningitis, Guillian Barre syndrome, rhabdomyolysis, AV-block, Takatzubo syndrome, acute myocardial infarction, congestive heart failure exacerbation, acute kidney injury, diabetic ketoacidosis, urinary tract infection, aspiration pneumonia, acute respiratory failure, fracture on admission, myocarditis, ventilation associated pneumonia, acute liver failure, mechanical ventilation, antibiotic treatment). Variables with p<0.08 were included in the multivariable analysis.

## Discussion

Influenza B was the predominant virus causing hospitalization due to RVI in our study during the winter of 2017–18, as compared with the 2 previous seasons. This predominance was evident throughout Israel, and the ICDC reported in July 2018, that out of 606 influenza positive samples taken in the sentinel clinics since October 1^st^ 2017, 70% were FluB, and 30% were FluA (55% H1 and 45% H3 strains)[11]. Influenza B/Yamagata strain, surpassing rates of FluA in this season, was also described in Europe [12]. In Israel, B/Victoria was dominant during 2015–16, estimated to infect 45% of the population of Israel [13], while in 2016–17, FluB was barely diagnosed (but included mainly B/Yamagata strain). In the 2017–18 season, B/Yamagata dominated. This lineage was covered only by the quadrivalent influenza vaccine (QIV), administered by only 2 out of 5 health maintenance organizations (HMOs) in Israel. This fluctuation in the appearance and disappearance of FluB lineages along several years was described in Israel during past winters[13]. Since most of the infection-aspects that were studied were similar between FluA and FluB, the 2017–18 FluB predominance (as well as its absence in the 2016–17 winter) did not affect the overall RVI severity. RSV, which was slightly more frequent in the 2017–18 season, may have contributed to RVI severity, since RSV-infected patients were older, sicker with significantly worse outcomes.

Apart for pandemics, there are no major epidemiological differences between FluA and FluB. Several studies have shown predilection of FluB for children and young adults [3, 14–16], but epidemiology data regarding hospitalized adults are less clear.

We have found only minor epidemiological differences between FluA and FluB, which included significantly lower Charlson scores and lower rates of IHD among FluB hospitalized patients. In our cohort, which included patients 16 years and older, there were no differences in the average age, hospitalization rates and lengths, comorbidities or other demographic features between the 2 viruses. An important epidemiological exception was the percentage of the vaccinated patients who were hospitalized with FluA vs FluB. Vaccination had a protective effect in preventing hospitalization for both viruses, represented by the significantly higher rates of hospitalized patients who were either non-vaccinated or their vaccination status was unknown, as compared to vaccinated patients. But while 14% reported receiving vaccine in the current season had documented FluA, only 3% of vaccinated patients were hospitalized due to FluB (p = 0.002). This suggests that influenza vaccine (trivalent and QIV) may have prevented hospitalization of FluB significantly better than it did for FluA. When analyzing the data according to the type of vaccine given, no patient (0/44) who received the QIV, and 4.8% (5/104) who received the trivalent vaccine were hospitalized with FluB. The respective figures regarding FluA were 14.9% and 13.2%. These data relate to the 3 seasons combined, i.e. including the winter of 2016–17 in which FluA dominated. Several other studies reported better vaccine effectiveness (VE) against FluB (as compared with FluA viruses),[17–20] but more data are needed in order to confirm these findings.

Clear epidemiological differences between RSV and influenza patients were seen in our study, including significantly older age with significantly higher Charlson score. Older age was probably the surrogate of significantly higher prevalence of IHD, CHF, rheumatic heart disease, chronic kidney disease, diabetes mellitus and dementia seen among the RSV cohort. This is in concordance with other studies describing the features of susceptible adults suffering from RSV [8, 9, 21–23]. Falsey et al [7] did not find epidemiological differences between RSV and influenza patients, but they enrolled only healthy elderly patients (>64 years of age), high-risk adults (having heart or lung disease) and patients hospitalized with acute cardiopulmonary conditions; as opposed to our cohort, which included all patients. Higher rates of patients with RSV were not seen among LTCF dwellers, representing the absence of LTCF-related RSV outbreaks in our cohort.

The clinical presentation of the 2 influenza viruses overlaps considerably. Although infections with FluB are sometimes considered less severe than those caused by A/H3N2 [24], several studies found either similar clinical features between the viruses [25–27], or only small differences, such as more gastrointestinal symptoms and myalgia among children with FluB when compared to FluA [28]. Surprisingly enough, comparative clinical presentation differences between FluA and FluB among hospitalized adults have not been thoroughly studied. In our study, hospitalized patients with FluB had more rhinorrhea and diarrhea than patients with FluA, and lower rates of dyspnea and stupor. Babcock et al reviewed the symptomatology of 207 hospitalized patients with influenza (186 FluA and 21 FluB) during 3 winter seasons and found no differences related to the influenza type [29]. Wie et al compared the clinical symptoms of laboratory confirmed FluA/H3N2 and FluB, among *non-hospitalized* adult patients during the 2011–12 season, and also found higher rates of rhinorrhea and gastrointestinal symptoms (nausea/vomiting, abdominal pain and diarrhea (although the latter did not reach statistical significance)) among FluB patients [20]. Weakness, headache and chest pain, that were also found to be more frequent in FluB in this study, were not seen in our cohort. Another study of non-hospitalized patients, found FluB to be associated with higher rates of fatigue and wheezing among adults, symptoms that were not addressed in our cohort [30]. Another large, sentinel-based study of non-hospitalized patients from France, found very minor clinical differences between the different influenza subtypes among adults [31].

As with the epidemiology, the considerable clinical presentation differences between influenza viruses and RSV have been described[32]. Differentiation between the influenza and RSV may be important since hospitalized patients with influenza should be treated early, while there is no current available treatment for RSV, and neuroaminidase inhibitors are not devoid of side effects. Our findings are in concordance with previously reported data [32]. Fever was noted in only 55% of patients in our cohort, while in influenza it was reported in over 80% (p<0.0001). Dyspnea and stupor were significantly more common among patients with RSV, which could represent the fact of being significantly older with higher rates of preexisting heart failure, dementia, kidney failure and other comorbidities. We found lower rates of headache, dizziness and falls among patients with RSV as compared with influenza, which may represent genuine pathologic differences between the viruses. Lower rates of fever and headache as compared with influenza were also noted among previously healthy working adults with RSV [33]. These clinical disparities, and especially the absence of fever and headache, could help clinicians to consider the possible diagnosis of RSV earlier (consideration not occurring instinctively among physicians treating adult patients).

Known risk factors for complications of both influenza and RSV include advanced age, preexisting cardiopulmonary, neurological, immunosuppression, and other common medical conditions [8, 34]. Complications occurred commonly among patients with both influenza and RSV in our cohort. Regarding influenza complications, we could not find any clinical-outcome differences between FluA and FluB. The equal role of FluB in causing morbidity and mortality as FluA among hospitalized patients was also described by Su et al [35] and in a systematic review [25]. The most common complications of influenza were bacterial infection, especially pneumonia (19%), acute kidney injury and CHF exacerbation. Acute respiratory failure was seen in 8% of patients, resulting in mechanical ventilation. Acute myocardial infarction (AMI) was evident in 6% and 2% of patients with FluA and FluB, respectively (20/454 patients). This association is consistent with a recently published study showing the higher risk-ratio for myocardial infarction after influenza infection [36]. Forty-four (9.2%) patients with influenza in our cohort presented with a fall, among which 6 (1.2%) had bone fractures. This complication was more prevalent during the 2017–18 season, where 24/167 (14%) patients with influenza fell, and 3% had fractures. Thirty influenza patients (6.6%) died during the index hospitalization, rates comparable to other studies.

RSV caused 15.7% of laboratory-confirmed RVI related hospitalization and patients with RSV had higher rates of bacterial complications than patients with influenza: one out of three patients had bacterial pneumonia, 14% had CHF exacerbations, 20% acute respiratory failure needing mechanical ventilation and 7% needed tracheostomy. Ivey et al reviewed the literature of cardiovascular complications of RSV among adults, and reported similar figures (13–20%) of CHF exacerbation[22]. In a significant part of infected patients (8% of influenza patients and 10% of RSV patients) PAF was documented on admission. PAF was recently found to be significantly more common among patients with influenza compared with those without [37], and high rates of arrhythmias were described also among patients with RSV [38–40]. Rhabdomyolysis and other neuromuscular complications seen in influenza were not encountered among RSV patients. Mortality rate of 13% among RSV patients was seen, probably signifying their pre-infection morbidity and older age. During the first 3 weeks of hospitalization, RSV infection resulted in significantly higher death rates than influenza, p = 0.019 (Fig 5). We believe that the burden on hospitalization rates as well as the morbidity and mortality (surpassing that of influenza), inflicted by RSV among hospitalized adults is considerably under recognized among treating physicians.

As expected, in a Cox regression model, older age was found to be a crucial prognostic factor for death, as well as previous hematologic diseases, and complications occurring during hospitalizations, mainly pulmonary, cardiac and renal failures. Cardiovascular complications associated with RSV were found to increase the risk of death also by others (hazard ratio 1.71)[39].

This study has limitations. First, as any retrospective study it has the limitations of inaccuracies probably present in the record files of patients, and it is subject to biases. Second, the study was conducted in a single institute with altogether limited number of subjects. Third, not all the patients hospitalized with RVI were PCR tested in a systematic fashion, a fact that could result in a bias towards including more severe cases in this study. Forth, we did not include a control group, and therefore we could not compare the epidemiologic characteristics of infected vs non-infected patients, and to calculate VE. Fifth, the data regarding the vaccination status were lacking, since in most cases it was not mentioned in the medical record, and so, only the cases that reported being vaccinated were probably valid, while patients in whom vaccination status was unknown from the files, could still have been vaccinated. This may have caused our conclusions regarding the efficiency of the vaccine to be over-estimated. Nevertheless, this potential bias should not have been related specifically to FluB, so the trends are still probably correct.

## Conclusions

In summary, we have reflected the predominance of FluB during the 2017–18 winter season occurring within and beyond the borders of Israel, while comparing it to FluA and RSV. We have confirmed the equivalence of FluB and FluA in many epidemiological and clinical aspects, including their role in causing severe disease among hospitalized adults. We demonstrated the immense impact of RSV on adult hospitalization rate, its characteristic susceptible population, unique clinical features (and differences from those of influenza), as well as its related poor outcomes, including death, surpassing those of influenza. By studying thoroughly the symptomatology of FluA and FluB, we were also able to contribute to a surprisingly unexplained lack of medical data regarding influenza symptomatology among hospitalized adults. High rates of complications such as AMI, PAF, falls and bone fractures related to these viruses may also contribute to complete the clinical picture of infected hospitalized adults with influenza and RSV infections.

## Supporting information

S1 Table ICD9 codes included for respiratory viral infection (RVI) related hospitalizations. (DOCX)

S2 Table Bacteria isolated from patients with viral-confirmed RVI. (DOCX)

S1 Dataset (XLSX)

## References

[1] Pretorius MA, Tempia S, Walaza S, Cohen AL, Moyes J, Variava E, et al The role of influenza, RSV and other common respiratory viruses in severe acute respiratory infections and influenza-like illness in a population with a high HIV sero-prevalence, South Africa 2012–2015. J Clin Virol. 2016;75:21–6. 10.1016/j.jcv.2015.12.004 26741826 PMC5712432

[2] Wang D, Chen L, Ding Y, Zhang J, Hua J, Geng Q, et al Viral etiology of medically attended influenza-like illnesses in children less than five years old in Suzhou, China, 2011–2014. J Med Virol. 2016;88(8):1334–40. 10.1002/jmv.24480 . 26792409 PMC7166643

[3] Paul Glezen W, Schmier JK, Kuehn CM, Ryan KJ, Oxford J. The burden of influenza B: a structured literature review. Am J Public Health. 2013;103(3):e43–51. 10.2105/AJPH.2012.301137 23327249 PMC3673513

[4] Adlhoch C, Snacken R, Melidou A, Ionescu S, Penttinen P, The European Influenza Surveillance N. Dominant influenza A(H3N2) and B/Yamagata virus circulation in EU/EEA, 2016/17 and 2017/18 seasons, respectively. Euro Surveill. 2018;23(13). 10.2807/1560-7917.ES.2018.23.13.18-00146 29616611 PMC5883452

[5] Sharabi S, Drori Y, Micheli M, Friedman N, Orzitzer S, Bassal R, et al Epidemiological and Virological Characterization of Influenza B Virus Infections. PLoS One. 2016;11(8):e0161195 10.1371/journal.pone.0161195 27533045 PMC4988634

[6] Control ICfD. https://www.health.gov.il/English/MinistryUnits/ICDC/Infectious_diseases/Flu/Pages/FWR.aspx. 2018.

[7] Falsey AR, Hennessey PA, Formica MA, Cox C, Walsh EE. Respiratory syncytial virus infection in elderly and high-risk adults. N Engl J Med. 2005;352(17):1749–59. 10.1056/NEJMoa043951 . 15858184

[8] Kestler M, Munoz P, Mateos M, Adrados D, Bouza E. Respiratory syncytial virus burden among adults during flu season: an underestimated pathology. J Hosp Infect. 2018 10.1016/j.jhin.2018.03.034 . 29614245

[9] Malosh RE, Martin ET, Callear AP, Petrie JG, Lauring AS, Lamerato L, et al Respiratory syncytial virus hospitalization in middle-aged and older adults. J Clin Virol. 2017;96:37–43. 10.1016/j.jcv.2017.09.001 28942341 PMC5889293

[10] Caram LB, Chen J, Taggart EW, Hillyard DR, She R, Polage CR, et al Respiratory syncytial virus outbreak in a long-term care facility detected using reverse transcriptase polymerase chain reaction: an argument for real-time detection methods. J Am Geriatr Soc. 2009;57(3):482–5. 10.1111/j.1532-5415.2008.02153.x . 19187415 PMC7166908

[11] Control ICfD. https://www.health.gov.il/PublicationsFiles/FLU_07042018.pdf. 2018.

[12] Control ECfDPa. Influenza virus characterization, summary Europe, https://ecdc.europa.eu/en/publications-data/influenza-virus-characterisation-summary-europe-june-2018. 2018.

[13] Sharabi S, Bassal R, Friedman N, Drori Y, Alter H, Glatman-Freedman A, et al Forty five percent of the Israeli population were infected with the influenza B Victoria virus during the winter season 2015–16. Oncotarget. 2018;9(5):6623–9. 10.18632/oncotarget.23601 29464098 PMC5814238

[14] Beaute J, Zucs P, Korsun N, Bragstad K, Enouf V, Kossyvakis A, et al Age-specific differences in influenza virus type and subtype distribution in the 2012/2013 season in 12 European countries. Epidemiol Infect. 2015;143(14):2950–8. 10.1017/S0950268814003422 25648399 PMC4595855

[15] Jennings L, Huang QS, Barr I, Lee PI, Kim WJ, Buchy P, et al Literature review of the epidemiology of influenza B disease in 15 countries in the Asia-Pacific region. Influenza Other Respir Viruses. 2018;12(3):383–411. 10.1111/irv.12522 29127742 PMC5907823

[16] Moa AM, Muscatello DJ, Turner RM, MacIntyre CR. Epidemiology of influenza B in Australia: 2001–2014 influenza seasons. Influenza Other Respir Viruses. 2017;11(2):102–9. 10.1111/irv.12432 27650482 PMC5304570

[17] Belongia EA, Simpson MD, King JP, Sundaram ME, Kelley NS, Osterholm MT, et al Variable influenza vaccine effectiveness by subtype: a systematic review and meta-analysis of test-negative design studies. Lancet Infect Dis. 2016;16(8):942–51. 10.1016/S1473-3099(16)00129-8 . 27061888

[18] Flannery B, Chung JR, Monto AS, Martin ET, Belongia EA, McLean HQ, et al Influenza Vaccine Effectiveness in the United States during the 2016–2017 Season. Clin Infect Dis. 2018 10.1093/cid/ciy775 . 30204854 PMC6522684

[19] Puig-Barbera J, Natividad-Sancho A, Launay O, Burtseva E, Ciblak MA, Tormos A, et al 2012–2013 Seasonal influenza vaccine effectiveness against influenza hospitalizations: results from the global influenza hospital surveillance network. PLoS One. 2014;9(6):e100497 10.1371/journal.pone.0100497 24945510 PMC4063939

[20] Wie SH, So BH, Song JY, Cheong HJ, Seo YB, Choi SH, et al A comparison of the clinical and epidemiological characteristics of adult patients with laboratory-confirmed influenza A or B during the 2011–2012 influenza season in Korea: a multi-center study. PLoS One. 2013;8(5):e62685 10.1371/journal.pone.0062685 23671624 PMC3643978

[21] Falsey AR, Walsh EE. Respiratory syncytial virus infection in elderly adults. Drugs Aging. 2005;22(7):577–87. 10.2165/00002512-200522070-00004 . 16038573 PMC7099998

[22] Ivey KS, Edwards KM, Talbot HK. Respiratory Syncytial Virus and Associations With Cardiovascular Disease in Adults. J Am Coll Cardiol. 2018;71(14):1574–83. 10.1016/j.jacc.2018.02.013 . 29622165

[23] Dowell SF, Anderson LJ, Gary HE Jr., Erdman DD, Plouffe JF, File TM Jr., et al Respiratory syncytial virus is an important cause of community-acquired lower respiratory infection among hospitalized adults. J Infect Dis. 1996;174(3):456–62. . 8769600 10.1093/infdis/174.3.456

[24] van de Sandt CE, Bodewes R, Rimmelzwaan GF, de Vries RD. Influenza B viruses: not to be discounted. Future Microbiol. 2015;10(9):1447–65. 10.2217/fmb.15.65 . 26357957

[25] Caini S, Kroneman M, Wiegers T, El Guerche-Seblain C, Paget J. Clinical characteristics and severity of influenza infections by virus type, subtype, and lineage: A systematic literature review. Influenza Other Respir Viruses. 2018 10.1111/irv.12575 . 29858537 PMC6185883

[26] Chagvardieff A, Persico N, Marmillot C, Badiaga S, Charrel R, Roch A. Prospective comparative study of characteristics associated with influenza A and B in adults. Med Mal Infect. 2018;48(3):180–7. 10.1016/j.medmal.2017.11.007 . 29258804

[27] Cohen JM, Silva ML, Caini S, Ciblak M, Mosnier A, Daviaud I, et al Striking Similarities in the Presentation and Duration of Illness of Influenza A and B in the Community: A Study Based on Sentinel Surveillance Networks in France and Turkey, 2010–2012. PLoS One. 2015;10(10):e0139431 10.1371/journal.pone.0139431 26426119 PMC4591015

[28] Hong KW, Cheong HJ, Song JY, Noh JY, Yang TU, Kim WJ. Clinical manifestations of influenza A and B in children and adults at a tertiary hospital in Korea during the 2011–2012 season. Jpn J Infect Dis. 2015;68(1):20–6. 10.7883/yoken.JJID.2013.466 . 25420662

[29] Babcock HM, Merz LR, Fraser VJ. Is influenza an influenza-like illness? Clinical presentation of influenza in hospitalized patients. Infect Control Hosp Epidemiol. 2006;27(3):266–70. 10.1086/501539 . 16532414

[30] Irving SA, Patel DC, Kieke BA, Donahue JG, Vandermause MF, Shay DK, et al Comparison of clinical features and outcomes of medically attended influenza A and influenza B in a defined population over four seasons: 2004–2005 through 2007–2008. Influenza Other Respir Viruses. 2012;6(1):37–43. 10.1111/j.1750-2659.2011.00263.x 21668663 PMC4941556

[31] Mosnier A, Caini S, Daviaud I, Nauleau E, Bui TT, Debost E, et al Clinical Characteristics Are Similar across Type A and B Influenza Virus Infections. PLoS One. 2015;10(9):e0136186 10.1371/journal.pone.0136186 26325069 PMC4556513

[32] Branche AR, Falsey AR. Respiratory syncytial virus infection in older adults: an under-recognized problem. Drugs Aging. 2015;32(4):261–9. 10.1007/s40266-015-0258-9 . 25851217

[33] Hall CB, Long CE, Schnabel KC. Respiratory syncytial virus infections in previously healthy working adults. Clin Infect Dis. 2001;33(6):792–6. 10.1086/322657 . 11512084

[34] Bennett JE, Dolin R, Blaser MJ. Mandell, Douglas, and Bennett's principles and practice of infectious diseases Eighth edition ed. Philadelphia, PA: Elsevier/Saunders; 2015. 2 volumes p.

[35] Su S, Chaves SS, Perez A, D'Mello T, Kirley PD, Yousey-Hindes K, et al Comparing clinical characteristics between hospitalized adults with laboratory-confirmed influenza A and B virus infection. Clin Infect Dis. 2014;59(2):252–5. 10.1093/cid/ciu269 . 24748521

[36] Kwong JC, Schwartz KL, Campitelli MA, Chung H, Crowcroft NS, Karnauchow T, et al Acute Myocardial Infarction after Laboratory-Confirmed Influenza Infection. N Engl J Med. 2018;378(4):345–53. 10.1056/NEJMoa1702090 . 29365305

[37] Chang TY, Chao TF, Liu CJ, Chen SJ, Chung FP, Liao JN, et al The association between influenza infection, vaccination, and atrial fibrillation: A nationwide case-control study. Heart Rhythm. 2016;13(6):1189–94. 10.1016/j.hrthm.2016.01.026 . 26850784

[38] Anderson NW, Binnicker MJ, Harris DM, Chirila RM, Brumble L, Mandrekar J, et al Morbidity and mortality among patients with respiratory syncytial virus infection: a 2-year retrospective review. Diagn Microbiol Infect Dis. 2016;85(3):367–71. 10.1016/j.diagmicrobio.2016.02.025 . 27179369

[39] Lee N, Lui GC, Wong KT, Li TC, Tse EC, Chan JY, et al High morbidity and mortality in adults hospitalized for respiratory syncytial virus infections. Clin Infect Dis. 2013;57(8):1069–77. 10.1093/cid/cit471 . 23876395

[40] Volling C, Hassan K, Mazzulli T, Green K, Al-Den A, Hunter P, et al Respiratory syncytial virus infection-associated hospitalization in adults: a retrospective cohort study. BMC Infect Dis. 2014;14:665 10.1186/s12879-014-0665-2 25494918 PMC4269936

