# Supplementary material for: Characteristics of hospitalized adult patients with laboratory documented Influenza A, B and Respiratory Syncytial Virus – A single center retrospective observational study
Source: PLoS One. 2019 Mar 28;14(3):e0214517. doi: 10.1371/journal.pone.0214517 (PMC6438521; doi:10.1371/journal.pone.0214517)
Supplement: S1 Table — (DOCX) [file pone.0214517.s001.docx]

| Diagnoses | ICD9 codes |
| --- | --- |
| Acute laryngitis | 464.0 |
| Bronchitis, not specified as acute or chronic | 490 |
| Acute bronchiolitis | 466.1 |
| Upper respiratory tract hypersensitivity reaction, site unspecified | 478.8 |
| aspiration pneumonia NOS | 507.0 |
| Asthma, unspecified | 493.9 |
| Bronchopneumonia, organism unspecified | 485 |
| Chronic obstructive pulmonary disease (COPD) | 496 |
| Cough | 786.2 |
| Dizziness and giddiness | 780.4 |
| Dyspnea and respiratory abnormalities | 786.0 |
| Fever, unspecified | 780.60 |
| Headache | 784.0 |
| Influenza, Influenza with pneumonia, With other respiratory manifestations | 487, 487.0, 487.1 |
| Pneumococcal pneumonia, Lobar pneumonia, organism unspecified | 481 |
| Pneumonia, organism unspecified | 486 |
| Myalgia and myositis, unspecified | 729.1 |
| Obstructive lung disease (and exacerbation) | 491.21 |
| Syncope and collapse | 780.2 |
| Respiratory Other | 786.09 |
| Acute respiratory failure, NOS | 518.81 |
| Other pulmonary insufficiency, not elsewhere classified | 518.82 |
| Acute upper respiratory infections of multiple or unspecified sites | 465 |
| Rhabdomyolysis | 728.88 |
| Sepsis, Severe sepsis, Septic shock | 995.91, 995.92, 785.52 |
| Acute sinusitis, unspecified | 461.9 |
| Stridor | 786.1 |
| Tachypnea | 786.06 |
| Throat pain | 784.1 |
| Acute pharyngitis, Acute tonsillitis | 462, 463 |
| Unspecified viral infection | 079.99 |
| Nausea with vomiting | 787.01 |
| Muscle weakness (generalized) | 728.87 |
| Wheezing | 786.07 |

S1 Table - ICD9 codes included for respiratory viral infections (RVI) related hospitalizations
