# Supplementary material for: Characteristics of hospitalized adult patients with laboratory documented Influenza A, B and Respiratory Syncytial Virus – A single center retrospective observational study
Source: PLoS One. 2019 Mar 28;14(3):e0214517. doi: 10.1371/journal.pone.0214517 (PMC6438521; doi:10.1371/journal.pone.0214517)
Supplement: S2 Table — (DOCX) [file pone.0214517.s002.docx]

S2 Table – Bacteria isolated from patients with viral-confirmed infection

| **Source** | **Bacterial type** | **Species** |
| --- | --- | --- |
| Blood stream isolates | 8 Streptococci | 3 *S. pneumoniae*, 2 GAS, 3 other streptococci |
|  | 5 non-fermenters | 3 *P. aeruginosa*, 2 *A. baumannii* |
|  | 1 Yeast | 1 *C. albicans* |
| Sputum isolates | 36 Gram positive cocci | 25 *S. auerus* (16 MSSA, 9 MRSA)  11 Streptococci (6 *S. pneumoniae*) |
|  | 28 Enterobacteriaceae | 13 *K. pneumoniae*, 6 *Enterobacter spp*, 9 others |
|  | 24 non-fermenters | 11 *P. aeruginosa*, 9 *A. baumannii*, 4 *S. maltophilia* |
|  | 8 *Haemophilus spp* | 7 *H. influenzae*, 1 *H. parainfluenzae* |
|  | 3 Filamentous fungi | 3 *Aspergillus spp*. |

RVI - Respiratory viral infection, GAS – Group A Streptococcus, MSSA – Methicillin sensitive *S. aureus*, MRSA – Methicillin resistant *S. aureus*
